# Infant Responses to Maternal Still Face at 9 Months Predict Social Abilities at 18 Months

**DOI:** 10.2188/jea.JE20090166

**Published:** 2010-03-05

**Authors:** Yuko Yato, Daisuke Tanaka, Ryoji Shinohara, Yuka Sugisawa, Emiko Tanaka, Lian Tong, Noriko Yamakawa, Tokie Anme, Masatoshi Kawai, Tadahiko Maeda, Japan Children’s Study Group

**Affiliations:** 1 Research Institute of Science and Technology for Society, Japan Science and Technology Agency, Tokyo, Japan; 2 Ritsumeikan University, College of Letters, Kyoto, Japan; 3 Tottori University, Tottori, Japan; 4 Graduate School of Comprehensive Human Sciences, University of Tsukuba, Tsukuba, Ibaraki, Japan; 5 Clinical Research Institute, Mie-chuo Medical Center, National Hospital Organization, Tsu, Japan; 6 Mukogawa Women’s University, Nishinomiya, Hyogo, Japan; 7 The Institute of Statistical Mathematics, Tokyo, Japan

**Keywords:** maternal still-face, social ability, longitudinal study, Interaction Rating Scale, Japanese children

## Abstract

**Background:**

This study investigated developmental change and stability in infant responses to the still-face (SF) situation, as well as predictive validity at age 18 months, focusing on autonomy and responsiveness.

**Methods:**

A total of 231 children (117 boys and 114 girls) and their Japanese mothers were observed in a face-to-face SF situation at two infant ages (4 and 9 months), as well as a caregiver-child teaching interaction at age 18 months. Each infant’s facial expression, gaze direction, and vocalization were coded according to the SF paradigm, including the Natural Interaction (NI) and SF phases. Each child’s Autonomy and Responsiveness to the Caregiver at age 18 months were both evaluated by means of the Interaction Rating Scale.

**Results:**

The results indicated that negative facial expression and vocalization in the SF phases at age 9 months predicted the Autonomy rating at age 18 months, while positive facial expression and gaze toward the caregiver in the NI at age 9 months predicted the Responsiveness to Caregiver rating at age 18 months.

**Conclusions:**

The results are discussed in the context of developmental continuity and change in the children’s social cognition and voluntary movements.

## INTRODUCTION

This study investigated developmental change and stability in responses to the still-face (SF) situation during the first year of life, and predictive validity for age 18 months, focusing on social abilities. Social abilities during childhood are fostered by complex interactions between a child’s temperament and environmental factors including family, peers, and other external entities. During the early years of life, the relationship between a child and a caregiver has great importance and such interactions form the foundation of the child’s ability to organize and respond to his/her world. A large amount of previous research has revealed that affectionate caregiver-child relationships during infancy predict social competence in later years.^1^^–^^3^

The SF situation, where a caregiver refrains from interacting with an infant and keeps his or her face neutral while maintaining silent eye contact, has been used to investigate infants’ responses to sudden changes in the emotional expressions of their caregivers.^4^ The SF situation is assumed to be one of the most robust procedures to test an infant’s recognition of social interaction patterns.

The still-face paradigm consists of two patterns: still-face phases; and natural interactions in the form of baseline and reunion. When caregivers become still-faced, some infants respond with a decreased positive affect (ie, less smiling or laughing) and an increased negative affect (ie, more fussing or crying), or by averting their gaze. However, other infants try to elicit their caregiver’s attention by means of positive affect and vocalization.^4^^–^^6^ These behaviors reflect an infant’s sensitivity to others’ facial expressions and contingency, in addition to his or her autonomy (namely, trying to initiate interaction with an unresponsive partner). Natural interactions (including baseline and reunion) are assumed to be suitable situations for evaluating responsiveness to a partner’s affectionate attention. Thus, infant facial expression, gaze direction, and vocalization in the SF paradigm are crucial indices of autonomy and responsiveness, both of which are important factors in the development of an infant’s social abilities.

We observed responses to the SF phases at 4 and 9 months of age, because newborn reflexes disappear during the interim between these two ages and infants rapidly develop social behaviors to express their inner states. They respond emotionally during face-to-face interaction,^7^^,^^8^ in addition to recognizing others as intentional agents.^9^ In other words, patterns of infant facial expression, gaze direction, and vocalization as predictors of later social behavior differ between ages 4 and 9 months; some of these social behaviors might be stable, but others not. Despite the fact that SF is one of the most appropriate ways to provoke the temperamental tendency in social ability with a moderate social stressor, few longitudinal studies have been conducted. Our study is somewhat innovative in that we traced developmental changes in infants’ responses to the SF situation and examined their predictive validity.

To assess a child’s autonomy and responsiveness in the second year of life, we observed caregiver-child teaching interaction at age 18 months and evaluated each child’s social-emotional autonomy and responsiveness toward the caregiver by means of the Interaction Rating Scale (IRS).^10^ We examined the predictive validity of the SF responses at ages 4 and 9 months by comparing them with the IRS scores at age 18 months.

To determine the factors that can predict individual differences in autonomy and responsiveness, the following hypotheses were formed. They are the basis for our examination of longitudinal relationships between infant response to caregiver’s SF (composed of repeated alternation of the natural-interaction (NI) phase and the SF phase) at ages 4 and 9 months, and IRS scores at age 18 months.

(1) Infants who respond to caregivers with a facial expression (positive or negative) and vocalization more frequently during the SF phases at ages 4 and 9 months would score higher on the IRS Autonomy scale at age 18 months.

Expressing emotions and vocalizing toward unresponsive caregivers is supposed to be a precursor of the social ability to initiate interaction spontaneously.

(2) Infants who respond to caregivers with positive facial expression, gaze, and vocalization more frequently during the NI phase at ages 4 and 9 months would score higher on the IRS Responsiveness to Caregiver scale at age 18 months.

Expressing positive affect, vocalizing, and looking at the caregiver during interaction are all supposed to be forms of responding to the caregiver’s emotions, vocalization, and eye contact.

Most of the research on children’s social abilities has been based on questionnaires assessed by their caregivers or teachers, or even the children themselves.^11^ In the current study, we utilized third-party observers to evaluate caregiver-child interaction during the first two years of life.

## METHODS

### Participants

Infants and adult caregivers were recruited from hospitals located in two nearby areas of central Japan (Mie and Osaka prefectures). All participants were videotaped in laboratory playrooms when the infants were 4, 9, and 18 months old. A total of 231 caregiver-child dyads (117 boys and 114 girls) were included in the final sample. Regarding educational background, 3.1% of the mothers had only gone as far as graduating junior high school, 26.7% had graduated high school only, 20.0% had completed technical college, and 50.2% were college/university graduates or had higher degrees. None of the babies had any serious medical complications.

This research was conducted in accordance with the Japan Science and Technology agency (JST) ethical committee standards regarding the treatment of participants.

### Procedures

In order to evaluate each child’s autonomy and responsiveness toward the caregiver, we investigated their responses to the maternal SF situation at ages 4 and 9 months, followed by their caregiver-child teaching interaction at age 18 months.

### Testing of still-face situation (4 and 9 months old)

Observations were conducted in a laboratory playroom (4.0 m × 4.0 m) equipped with a one-way mirror. The playrooms in both prefectures were identical: each was equipped with seven VTR cameras, a microphone, and an infant seat at the center of the room. Each caregiver sat on the floor in front of her infant, who was in the infant seat. The caregiver and infant were videotaped via synchronized cameras. The experiment was conducted while the infants were alert (ie, eyes open and attention directed toward external stimuli).

In the natural interaction (NI) phase, each caregiver was asked to interact with her infant in an ordinary manner, just as she normally did at home. In the SF phase, each of the caregivers was asked to freeze her face while gazing at her infant, to stop talking, and to refrain from any movement. The sequence of interactions was as follows: (1) natural interaction phase (NI1) for 30 seconds; (2) still-face phase (SF1) for 1 minute; (3) another natural interaction phase (NI2) for 30 seconds; and (4) another still-face phase (SF2) for 30 seconds. The order and duration of these phases was the same for all participants.

An experimenter who was positioned behind the infant seat signaled to the caregiver when to shift phase by waving his/her hand.

#### Measurements

Infants’ behaviors during the natural interaction phases of the procedure were coded in three separate dimensions based on the videotapes: Positive Facial Expression; Gaze at Caregiver; and Vocalization. Infants’ behaviors during the still-face phases were coded in three separate dimensions: Positive Facial Expression; Negative Facial Expression; and Vocalization.

These behaviors were coded by utilizing a five-second, one-zero time sampling method and the percentage of occurrences (ie, the ones) were calculated for each dimension in each phase. Coding for facial expressions was coded according to the MAX coding system.^12^

#### Coding and reliability

Coders who were unaware of the hypotheses viewed video records of the 150-second procedure and coded each dependent measure by pressing a button on a computer keyboard, thereby activating a computerized event recorder.

The videotapes were coded by graduate students, who were trained by one of the authors, and calibrated until an inter-rater reliability of .75 or more (Cohen’s kappa) was attained. In addition, 20% of the sessions were selected at random for independent coding (corroboration) by different coders. Reliability remained high across all dimensions and phases. The mean reliability coefficient was above .85 for each dimension.

### Assessment of social skills (18 months old)

Each dyad was observed in the same laboratory playroom where they had been observed when the child was 4 and 9 months of age. A chair and table was placed in the centre of the room. Each child was seated on the chair and each caregiver was seated on the floor in front of the child. Caregiver and child were videotaped via synchronized cameras.

Each caregiver was asked to teach her child a prescribed task using building blocks, which was slightly difficult to accomplish at that age. After the task, the caregiver was asked to tidy up the blocks with her child. The observation lasted for 1–5 minutes.

#### Measurements

The Interaction Rating Scale^10^ contains a well-developed set of observable behaviors that describe caregiver-child communication and interaction during a teaching situation. The scale consists of 70 items organized into 10 subscales. In our study, two of the IRS child subscales (Autonomy, Responsiveness to Caregiver) were analyzed in relation to our hypotheses. Each item was observed for each child to determine whether the behavior occurred or not, and the total number of binary zeroes (no occurrence of behavior) was then calculated for each item in the two subscales.

Items in the Autonomy subscale consist of statements which describe behaviors for initiating interaction with a caregiver, and items in the Responsiveness to Caregiver scale include the items to check verbal and nonverbal responses to caregivers.

#### Coding and reliability

Students other than those who rated the SF situation were recruited as coders. Each child behavior was coded separately by two trained coders who were unaware of the hypotheses. Coders needed to be sensitive to the cues of both the child and caregiver during the dyadic interaction, because the cues of young children are often ambiguous and subtle. In order to maintain satisfactory standards for the quality of data, we trained graduate students for over a month on how to code the videotapes of caregiver-child interaction. Each coder was trained until a minimum of 80% inter-rater agreement on pilot tapes was achieved. To corroborate each coder’s reliability, two coders examined and recoded 25% of the tapes, which were selected at random, and agreement surpassed 87%.

## RESULTS

**Figure 1.**
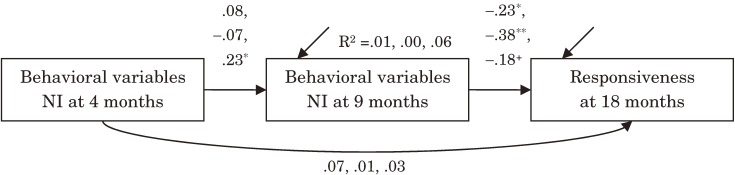
Results of Path analyses concerning behavioral variables during NI phase of SF procedure at age 4 and 9 months and Responsiveness to caregiver at age 18 months. Values represent estimates of standardized regression weights, R^2^, coefficient of determination, with respect to Positive Facial Expression, Gaze at Caregiver, and Vocalization, respectively. *P < .05, **P < .01, ^+^*P* < .10.

To examine our hypotheses, we employed path analyses which followed the chronological structure of the hypotheses. To investigate whether variables extracted from the NI phases of our procedure at ages 4 and 9 months could predict Responsiveness to Caregiver at age 18 months, we performed three independent path analyses (Table 1, Figure 1). Positive Facial Expression, Gaze at Caregiver, and Vocalization were used as explanatory variables (dimensions) at ages 4 and 9 months. These variables represent responsiveness in the SF framework as a factor of sociability. The path model used in this study was a full path model constrained by ordering according to age (number of months).

**Table 1.** Descriptive statistics of variables entered path analyses

	Behavioral variables	4 months		9 months		IRS child subscales	18 months	
			
		mean	SD	mean	SD		mean	SD
	
NI phases	Positive Facial Expression	0.9279	0.675 14	0.7846	0.584 78	Responsiveness	0.7446	1.0256
	Gaze at Caregiver	1.4571	0.457 72	1.2385	0.457 95			
	Vocalization	0.4633	0.484 39	0.6154	0.516 73			
	
SF phases	Vocalization	0.7517	0.569 20	0.9555	0.553 23	Autonomy	0.8225	1.1604
	Negative Facial Expression	0.6072	0.561 01	0.4034	0.472 04			
	Positive Facial Expression	0.4393	0.464 85	0.3788	0.378 31			

For Positive Facial Expression during the NI phases, the path from the occurrence rate at age 9 months to the responsiveness score at age 18 months was significant and the estimate of standardized regression weight was −.23 (*P* = .012). There were no other significant paths in this model. For Gaze at Caregiver during the NI phases, the path from the occurrence rate at age 9 months to the responsiveness score at age 18 months was significant and the estimate of standardized regression weight was −.38 (*P* < .001). There were no other significant paths in this model. In regard to Vocalization during the NI phases, however, there was only marginal significance between the occurrence rate at age 9 months and the responsiveness score at age 18 months (the estimate of standardized regression weight was −.18, *P* = .064); instead the path between ages 4 and 9 months (the occurrence rates) was significant and the estimate of standardized regression weight was .23 (*P* = .023).

**Figure 2.**
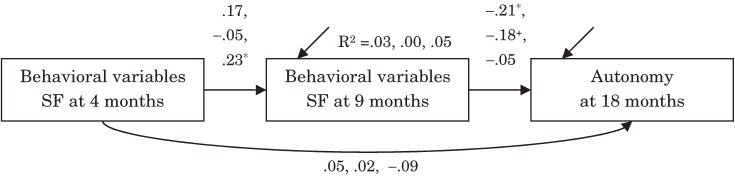
Results of Path analyses concerning behavioral variables during SF phase of SF procedure at age 4 and 9 months and Autonomy score at age 18 months. Values represent estimates of standardized regression weights, R^2^, coefficient of determination, with respect to Vocalization, Negative Facial Expression, and Positive Facial Expression, respectively. *P < .05, ^+^*P* < .10.

Next, we performed three independent path analyses to investigate whether variables extracted from the SF phases of our procedure at ages 4 and 9 months could predict Autonomy at age 18 months (Table 1, Figure 2). Positive Facial Expression, Negative Facial Expression, and Vocalization were extracted from the SF phases at ages 4 and 9 months and used as explanatory variables. These variables represent autonomy as behaviors based on an infant’s motivation to elicit emotional facial expression from a caregiver who presents an emotionless face. The model used in this analysis was the same as in our previous analyses.

For Vocalization during the SF phases, the path from the occurrence rate at age 9 months to the autonomy score at age 18 months was significant and the estimate of standardized regression weight was −.21 (*P* = .024). There were no other significant paths in this model. Negative Facial Expression showed the same pattern as Vocalization, but its path from the occurrence rate at age 9 months to the autonomy score at age 18 months was marginally significant and the estimate of standardized regression weight was −.18 (*P* = .055). There were no other significant paths in this analysis. Compared with these results, the Positive Facial Expression analysis reveals significant coherence between its occurrence rates at ages 4 and 9 months, and the estimate of standardized regression weight was .23 (*P* = .037).

## DISCUSSION

We observed infant responses to still-face interaction (alternating NI and SF phases) at ages 4 and 9 months, and caregiver-child interaction at age 18 months.

Our study revealed that some of the infant behaviors at age 9 months, but not 4 months, predicted autonomy and responsiveness at age 18 months. These findings suggest that the mechanism of facial expression, gaze direction, and vocalization as precursors of autonomy and responsiveness develop between the two aforementioned ages. In retrospect, we realize that there might be a limitation in the availability of these behavioral features at age 4 months to serve as potential precursors of social behaviors measured in the second year of life.

Positive facial expression (ie, smiling and laughing) and gaze as responses to the caregiver at age 9 months during natural interaction (NI) predicted responsiveness at age 18 months, but spontaneous smiling and laughing at still-faced (SF) caregivers did not predict autonomy at age 18 months. Instead, expressing negative affect during SF at age 9 months predicted autonomy at age 18 months.

Because the SF situation is mildly stressful for infants, expressing negative emotion is a reasonable response. In this light, properly communicating what they are feeling (eg, angry or fussy) is an important aspect of social abilities. Stenberg and Campos investigated the development of anger expression patterns and showed that infant facial expressions become more organized, and vocalization shows greater coordination with the face, at around age 4 months.^13^ Therefore, the 4-month-olds in our study probably had limited voluntary coordination skills for expressing negative emotion, and that might be the reason that only negative facial expression at age 9 months (in the SF phases) predicted autonomy at age 18 months. Our coding categories did not distinguish between negative and positive vocalizations in the SF phases, because these types of vocalizations were so difficult to discriminate from each other that inter-rater reliability was too low. However, we might conjecture that vocalization at age 9 months coordinates better with negative facial expression at that age, and thus can predict autonomy at age 18 months.

On the other hand, smiling and laughing at unresponsive caregivers during a still-face phase are ways of soliciting maternal response to restore natural interaction. Our findings revealed that positive facial expression in the still-face situation had stability between ages 4 and 9 months but it did not predict autonomous behavior at age 18 months. Spontaneous smiling in a socially stressful situation might differ in developmental mechanism from spontaneous social behavior during interaction with a responsive partner in the second year of life.

Our study revealed that infant gaze at caregiver was significant only at age 9 months as a predictor of responsiveness at age 18 months. This result could be explained in terms of the development of voluntary eye movements and emotional preference for caregivers.

Younger infants have difficulty disengaging gaze fixation from salient central stimuli, and their ability to make voluntary saccades across wide arcs is restricted.^14^^,^^15^ However, during the second half of the first year, infants shift their attention more fleetingly and intentionally.^16^ Moreover, caregiver-child attachment becomes clearer, with infants uniquely preferring their relationship with caregivers after age 6 months or so.^17^ It is likely that the 9-month-olds in our study gazed at their caregivers with more affection during the NI phases, which predicted responsiveness age 18 months of age.

In sum, behaviors at age 9 months, rather than 4 months, predict social abilities at age 18 months. The differential relationship between the two infant ages utilized in our study reflects developmental changes in social cognition, motor skills, and communication. Around the middle of the first year, expression of basic emotions, vocalization, and gesture all become well-organized and correspond meaningfully with environmental events,^6^^,^^18^ which follows that infants become more skillful in displaying their internal states.

Regarding IRS scores at age 18 months, items in the Autonomy subscale consist of statements which describe behaviors for initiating interaction with a caregiver, such as vocalizing, smiling, or eye-to-eye contact. The Responsiveness to Caregiver subscale includes items related to verbal and nonverbal responses to caregivers. In sum, behaviors evaluated by the IRS reflect a child’s ability to communicate through facial expression, gaze direction, and vocalization, which was coded in the SF situation at ages 4 and 9 months. The fact that infant behaviors observed during only 150 seconds (total time combining the NI and SF phases) can predict child behaviors in a naturalistic interaction nine months later is remarkable. Our results regarding the relationship between the SF and IRS paradigms lend some validity to both SF and IRS as useful measures of children’s social abilities.

## APPENDIX

### Japan Children's Study Group

Chairman: Zentaro Yamagata (Department of Health Sciences, School of Medicine, University of Yamanashi), Hideaki Koizumi (Advanced Research Laboratory, Hitachi, Ltd.).

Participating Researchers: Kevin K. F. Wong, Yoko Anji, Hiraku Ishida, Mizue Iwasaki, Aya Kutsuki, Misa Kuroki, Haruka Koike, Daisuke N Saito, Akiko Sawada, Yuka Shiotani, Daisuke Tanaka, Shunyue Cheng, Hiroshi Toyoda, Kumiko Namba, Tamami Fukushi, Tomoyo Morita, Hisakazu Yanaka (Research Institute of Science and Technology for Society, Japan Science and Technology Agency), Yoichi Sakakihara (Department of Child Care and Education, Ochanomizu University), Kanehisa Morimoto (Graduate School of Medicine, Osaka University), Kayako Nakagawa (Graduate School of Engineering, Osaka University), Shoji Itakura (Graduate School of Letters, Kyoto University), Kiyotaka Tomiwa (Graduate School of Medicine, Kyoto University), Shunya Sogon (The Graduate Divisiton of the faculty of Human Relations, Kyoto Koka Women’s University), Toyojiro Matsuishi (Department of Pediatrics and Child Health, Kurume University), Tamiko Ogura (Graduate School of Humanities, Kobe University), Masako Okada (Koka City Educational Research Center), Hiroko Ikeda (National Epilepsy Center Shizuoka Institute of Epilepsy and Neurological Disorder), Norihiro Sadato (National Institute for Physiological Sciences, National Institutes of Natural Sciences), Mariko Y. Momoi, Hirosato Shiokawa, Takanori Yamagata (Department of Pediatrics, Jichi Medical University), Tadahiko Maeda, Tohru Ozaki (The Institute of Statistical Mathematics, Research Organization of Information and Systems), Tokie Anme (Graduate School of Comprehensive Human Sciences, University of Tsukuba), Takahiro Hoshino (Graduate School of Arts and Sciences, The University of Tokyo), Osamu Sakura (Interfaculty Initiative in Information Studies, The University of Tokyo), Yukuo Konishi (Department of Infants’ Brain & Cognitive Development, Tokyo Women’s Medical University), Katsutoshi Kobayashi (Center for Education and Society, Tottori University), Tatsuya Koeda, Toshitaka Tamaru, Shinako Terakawa, Ayumi Seki, Ariko Takeuchi (Faculty of Regional Sciences, Tottori University), Hideo Kawaguchi (Advanced Research Laboratory, Hitachi, Ltd.), Sonoko Egami (Hokkaido University of Education), Yoshihiro Komada (Department of Pediatric and Developmental Science, Mie University Graduate School of Medicine Institute of Molecular and Experimental Medicine), Hatsumi Yamamoto, Motoki Bonno, Noriko Yamakawa (Clinical Research Institute, Mie-chuo Medical Center, National Hospital Organization), Masatoshi Kawai (Institute for Education, Mukogawa Women’s University), Yuko Yato (College of Letters, Ritsumeikan University), Koichi Negayama (Graduate School of Human Sciences, Waseda University).
